# Evidence of Non-microtubule Spindle Forces in *Mesostoma ehrenbergii* Spermatocytes

**DOI:** 10.3389/fmolb.2020.557990

**Published:** 2020-11-19

**Authors:** Eleni Fegaras-Arch, Michael Berns, Arthur Forer

**Affiliations:** ^1^Department of Biology, York University, Toronto, ON, Canada; ^2^Departments of Biomedical Engineering and Cell Biology, Beckman Laser Institute, University of California, Irvine, Irvine, CA, United States; ^3^Institute for Engineering in Medicine and Department of Bioengineering, University of California, San Diego, La Jolla, CA, United States

**Keywords:** meiosis, *Mesostoma*, laser irradiation, microtubules, spindle matrix

## Abstract

We tested conclusions reached in previous experiments in which *Mesostoma* spermatocyte chromosomes moved rapidly to a pole in the absence of microtubules: after 10 μM nocodazole (NOC) depolymerized metaphase spindle microtubules, kinetochores from each of the 3 bivalents detached from the same pole and rapidly moved to the other pole, at speeds averaging 37.7 μm/min. with some as high as 100 μm/min. We concluded that these very fast movements were due to non-microtubule forces arising from a spindle matrix. However, since the chromosomes stretch out before detaching, there is tension in the chromosomes from the stretch. Thus the movements of detached kinetochores conceivably might be due to recoil from the tension, though we argued against this possibility (Fegaras and Forer, 2018a). In this article we test whether recoil causes the movements. We cut bivalents into 2 pieces, using a femtosecond laser, before addition of NOC. When 1 bivalent was severed, all kinetochores moved to one pole in 12/15 cells; when 2 bivalents were severed, all kinetochores moved to one pole in 4/6 cells; and when all 3 bivalents were severed all kinetochores moved to one pole in 3/9 cells. The bivalent “halves” moved rapidly, with average speeds of 47 μm/min, velocities that are not significantly different from those in cells without any laser-cut bivalents (*p* > 0.05). Since kinetochores move at the same speeds whether they are part of bivalents or not, NOC-induced chromosome movements are not due to recoil from tension along the full-length bivalent, strongly supporting the idea that non-microtubule forces move chromosomes in *Mesostoma* spermatocytes.

## Introduction

The present work studies the mechanism by which chromosomes move rapidly in the absence of microtubules in *Mesostoma* spermatocytes. Before we describe our present experiments we summarize some important features of meiosis-I in *Mesostoma* spermatocytes.

**FIGURE 1:**
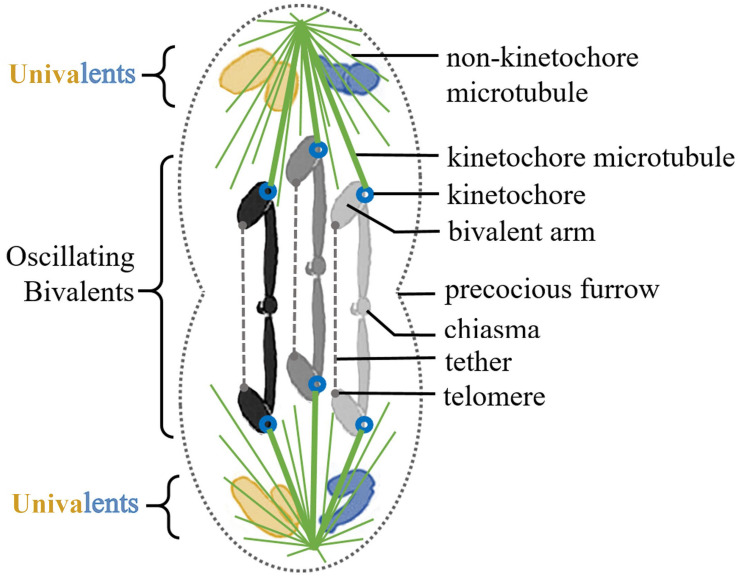
Illustration of a *Mesostoma* primary spermatocyte. There are two univalent pairs, acrocentrics in blue and metacentrics in yellow. There are three metacentric bivalents, in gray scale. Each bivalent has a tether extending between the telomeres of their free arms. The microtubule spindle is in green, with thick kinetochore microtubules extending from bivalent kinetochores to the two poles. The precocious cleavage furrow is illustrated as slightly ingressed at the midline of the cell.

*Mesostoma* spermatocytes have three bivalent chromosomes and two sets of univalent chromosomes positioned at either pole, as first described in Husted and Ruebush, 1940 (Figure 1). A precocious pre-anaphase cleavage furrow develops and ingresses slightly during early prometaphase giving the cell its characteristic dumbbell-like shape, then recommences ingression during anaphase at which time it cleaves the cell in the usual manner (Forer and Pickett-Heaps, 2010; Fegaras and Forer, 2018b). The univalent chromosomes are never paired: there are only 3 synaptonemal complexes in pre-division nuclei, corresponding to each of the 3 bivalents (Oakley and Jones, 1982). Throughout prometaphase the three bivalent chromosomes oscillate toward and away from the two poles with excursion distances averaging 4 μm and at speeds averaging 5–6 μm/min (e.g., Ferraro-Gideon et al., 2014). The chromosomes never form a metaphase plate: oscillations continue until anaphase. At anaphase the bivalent oscillations end abruptly and the segregating chromosomes move toward the two poles at speeds of approximately 1 μm/min (Fuge, 1987, 1989; Ferraro-Gideon et al., 2013, 2014). For clarity, we must describe several other unusual behaviors in these cells.

Elastic tethers that extend between the separating arms of all anaphase chromosomes in a variety of cells also extend between the arms of separating *Mesostoma* spermatocyte anaphase chromosomes (Forer et al., 2017). Tether elasticity is shown when one cuts an arm: the arm fragment that is produced moves rapidly, telomere toward telomere, to the partner chromosome moving to the other pole. Tethers are elastic early in anaphase, but become inelastic later in anaphase as they elongate (e.g., LaFountain et al., 2002; Forer et al., 2017; Kite and Forer, 2020). In *Mesostoma* spermatocytes each bivalent has one chiasma and 2 free arms that are associated with each kinetochore, and similar elastic tethers extend between the free arms of the bivalents in prometaphase: when one cuts an arm, the arm fragment that is formed moves rapidly, telomere to telomere, to the free arm of the partner half-bivalent (Forer et al., 2017). This does not happen all the time in *Mesostoma*, but only about half the time. Absence of arm fragment movement indicates that either the tethers are inelastic at that time, or not present.

The anaphase chromosomes in *Mesostoma* spermatocytes may be distributed non-randomly to the daughter cells, i.e., male-derived chromosomes to one pole and female-derived chromosomes to the other. The first line of evidence suggesting this derives from detailed study of univalent chromosome movements. In early prometaphase there often are “faulty” distributions of univalents at the two poles but by anaphase there is one of each kind at each pole (Oakley, 1983, 1985). Univalents move between poles in living cells (Oakley, 1985; Forer and Pickett-Heaps, 2010; Ferraro-Gideon et al., 2014). Proper distribution of univalents seems to arise from their movements between the poles because early in prometaphase the univalents often are distributed wrongly (Oakley, 1983, 1985), such as 3 or 4 at one pole and one or none at the other, or two of one kind at one pole and two of the other kind at the other pole. Since the univalents often change poles more times than necessary to achieve the goal of one of each kind at each pole, in particular, “partner” univalents often switch poles after proper distribution has been achieved (Oakley, 1985), Oakley suggested that there is non-random segregation in these cells in that male-derived chromosomes go to one pole and female-derived chromosomes go to the other (Oakley, 1985): the “gratuitous” univalent excursions are due to sorting out male-derived from female-derived univalents. A second line of evidence that segregation may be non-random is that bipolar oriented bivalents frequently reorient (Ferraro-Gideon et al., 2014; Brady and Paliulis, 2015). One kinetochore releases from one pole, moves to the other pole, and then the other kinetochore moves to the vacated pole (Ferraro-Gideon et al., 2014). That is, the reorientations give rise to segregation to poles different from the original orientation. This may be because the bipolar orientation must segregate male-derived from female-derived chromosomes. A third line of evidence, that we now describe, is from experiments that we follow up on in this article, in which all chromosomes consistently move to one pole after addition of nocodazole (NOC) to depolymerize microtubules.

When microtubules are depolymerized in *Mesostoma* spermatocytes, the bivalents stop mid-oscillation and the sister kinetochores of each half-bivalent move toward their respective poles (Fegaras and Forer, 2018a). This causes the bivalents to stretch, possibly due to non-microtubules associated force production via the spindle matrix and/or actin-myosin. After a few minutes being stretched, all the kinetochores oriented to one pole detach and move rapidly toward the opposite pole, at speeds averaging 38 μm/min and with instantaneous speeds up to 100 μm/min (Fegaras and Forer, 2018a,b). One reason that all kinetochores move to the same pole might be because this is related to non-random segregation, because all male partners segregate to one pole and all females to the other.

The movements to the one pole after NOC treatment occurred in the absence of microtubules; Fegaras and Forer (2018a) argued that movements in the absence of microtubules were due to forces arising from the spindle matrix. But because the chromosomes stretch out and elongate by 25–30% of their original length before detaching, the rapid chromosome movement conceivably may be due to a release in tension along the length of the bivalent. Fegaras and Forer (2018a) argued against this interpretation for various reasons, including that the movements are not linear. We tested this point directly in experiments reported herein. We used a femtosecond laser to cut 1, 2, or 3 bivalents (per cell) into two pieces, two “halves.” After treatment with NOC, “halved” bivalents at one pole moved toward the other pole at the same rapid speeds that non-severed chromosomes move at. Since the movements occur in the absence of microtubules and in the absence of tension along the length of the chromosomes, we suggest that the chromosomes are moved by spindle matrix components such as actin and myosin.

While the focus of our experiments is on the question of what produces force for movement in the absence of microtubules, some of the results extend to the issue of why all kinetochores detach from one pole and move to the other, instead of detaching from poles at random, as they do in other cells (Pickett-Heaps and Spurck, 1982; discussed in Fegaras and Forer, 2018a), and to the issue of how the movements of all the kinetochores are coordinated. Some of our experimental manipulations altered the coordinated movements to one pole and implicated the tethers as being important in that coordination, thereby providing some insight into how the coordinations might occur.

## Materials and Methods

### Living Cell Preparations and Drug Addition

The procedures have been described in detail elsewhere (Fegaras and Forer, 2018a,b). In brief, *Mesostoma ehrenbergii* were reared in lab (Hoang et al., 2013). Testes were extracted from individual animals using pulled 5, 10, or 15 μL micropipettes (Fisher), and then expelled onto a coverslip into *Mesostoma* Ringers solution (61 mM NaCl, 2.3 mM KCl, 0.5 mM CaCl_2_, and 2.3 mM phosphate buffer) that contained 0.2 mg/mL fibrinogen (Calbiochem). Thrombin was added to the fibrinogen to hold the cells in a fibrin clot and the cells in the clot were then immersed in *Mesostoma* Ringers solution in a perfusion chamber (Forer and Pickett-Heaps, 2005). For drug treatment, cells were perfused with 10 μM nocodazole (NOC, from Sigma) a microtubule depolymerizing agent (Vasquez et al., 1997; Jordan and Wilson, 2004; Poxleitner et al., 2008), in *Mesostoma* Ringers: NOC from a 1000x concentrated stock in dimethyl sulfoxide (DMSO) was diluted with *Mesostoma* Ringer’s solution to reach the desired concentration. Cells were viewed with phase-contrast microscopy using a 63x, NA 1.4, Zeiss Plan Apochromatic Lens. We recorded live cells, captured digital images every 2–3 s, and cropped the images and time-stamped them (with data from the recorded file images) using the freeware program IrfanView. Time-lapsed sequences and avi files were made using freeware Virtualdub2, and movement graphs were obtained using our in-house program WinImage (Wong and Forer, 2003), in which the position of each kinetochore was plotted relative to a fixed point at the cell edge. Chromosome movement graphs were generated using the program SlideWrite 7.0. We determined chromosome speeds from computer generated lines-of-best fit to the points on the movement graphs. Movement graphs of kinetochore positions vs time appeared as sawtooth waves. We considered one complete bivalent oscillation as the distance from peak-to-peak or trough-to-trough of the sawtooth waves. We considered the distance along the *y*-axis between successive trough and peak as the amplitude, representing the distance a kinetochore travels during an oscillation. And we considered the distance along the *x*-axis between successive troughs or peaks as the period, representing the time a bivalent takes to complete an oscillation. Student’s *t*-tests were performed when comparing chromosome speeds.

### Laser Irradiations

**FIGURE 2:**
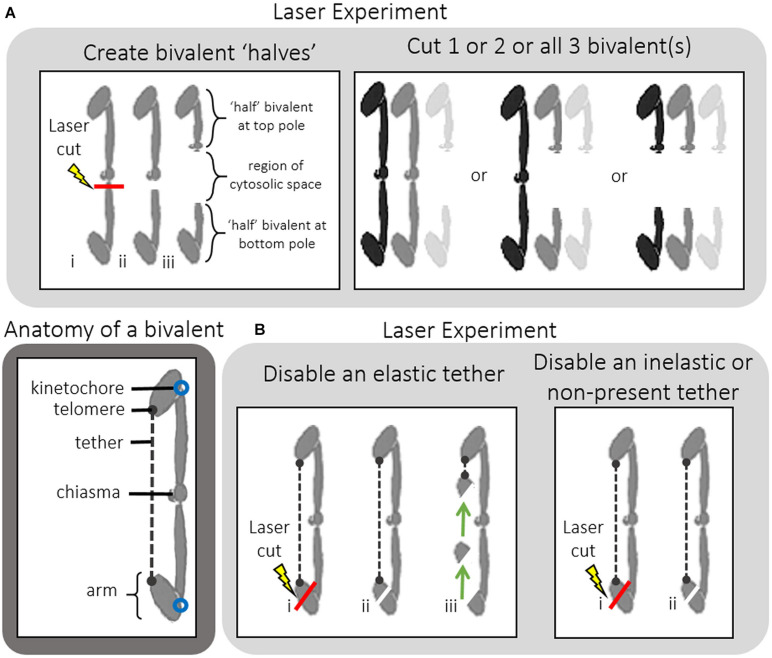
**(A,B)** Anatomy of a bivalent and a guide to the two types of laser cuts performed on bivalents. **(A)** *Creating bivalent “halves*.” Laser irradiations of bivalent chromosomes were performed near the middle of the chromosome, near the chiasma; we refer to the resultant pieces as “halves,” or “halved bivalents” because bivalents are not necessarily cut precisely in half. Successful severing of the bivalents was determined by observing a region of cytosolic space between the two bivalent “halves.” Either 1, 2, or all 3 bivalents were cut in half within any one given cell, after which NOC was added. **(B)** *Disabling tethers*. Tethers extend between the telomeres on the free arms of each bivalent. In order to functionally disable them a portion of the arm near the tip of one bivalent arm was cut off. If the arm fragment moved toward the opposite arm (as shown by the arrows) this indicated the tether was elastic. If the arm fragment did not move this indicated the tether was inelastic. NOC was then added to these cells.

Spermatocytes were observed using phase-contrast microscopy. User-defined positions were irradiated using a 200 fs-pulsed laser (Mai Tai, Newport Co., Irvine, CA, United States) that emitted 740 nm wavelength light. System details can be found in Berns and Greulich, 2007; Harsono et al., 2012; Forer et al., 2017. We recorded digital images every 2 s and performed cuts in three different planes of focus along the *Z*-axis. Bivalents were cut in two different regions: near the chiasma of the bivalent to create bivalent “halves” or near the telomere of a bivalent arm to disable tethers (Figure 2). Laser irradiations of bivalent chromosomes were performed near the middle of the chromosome, near the region of the chiasma; we refer to the resultant pieces as “halves,” or “halved bivalents” because bivalents are not necessarily cut precisely in half. Sometimes cutting a bivalent required more than one series of cuts. During prometaphase oscillations the two kinetochores on one normal bivalent sometimes move in the same direction (i.e., one moves toward its pole and the other moves away from its pole). Sometimes the two kinetochores move in opposite directions (each toward its own pole). We cut bivalents most often when the bivalent kinetochores moved toward opposite poles because chromosomes are more stretched out then and because kinetochore movements tend to briefly pause for a few seconds once they reach a pole, making the “moving target” more stationary (Ferraro-Gideon et al., 2014). We compared oscillations of “halved” bivalents with those of non-cut bivalents by comparing the saw-tooth waves of movement. We considered “half” bivalent oscillations as normal when they have the same oscillation period and velocities as before laser treatment. We considered “half” bivalent oscillations as irregular when they changed 50% or more in period and/or velocity. In cells treated with both NOC and laser cuts, bivalents were cut up to 15 min before NOC was added.

## Results

### Control Cells and NOC Treated Cells

During prometaphase-I in *Mesostoma* spermatocytes, the three bivalents continuously oscillate toward and away from either pole. When cells are treated with 10 μM NOC the bivalent chromosomes immediately stop oscillating and each kinetochore moves toward its respective pole as the bivalent stretches out. The chromosomes pause (i.e., remain stretched out) and after a few minutes all three kinetochores at one pole detach and move toward the opposite pole, after which the cleavage furrow moves toward the vacated half spindle (Fegaras and Forer, 2018a,b).

### Bivalent Chromosomes “Halved” With a Laser

#### Effect of Severing Bivalent(s) Prior to Treatment With NOC

We first describe the behavior of the “halved” bivalents, both as aid to explaining the effects after NOC treatment and because of the relevance of their behavior to the possible non-random segregation in these cells.

**FIGURE 3:**
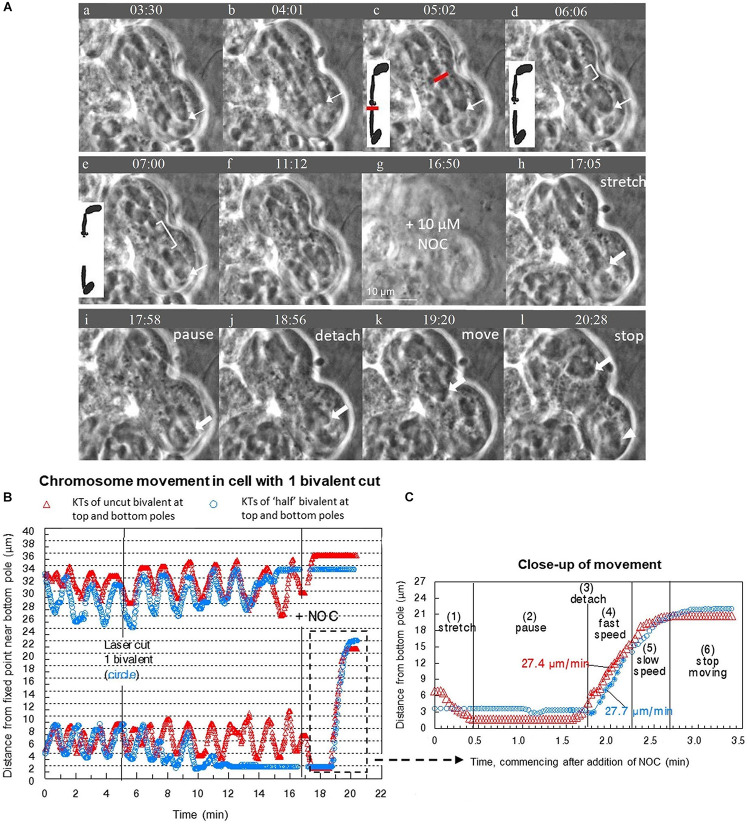
**(A–C)** After cutting 1 bivalent into two pieces during prometaphase, severed and unsevered bivalents still move after adding NOC to *Mesostoma* spermatocytes. **(A)** Image sequence where one bivalent is cut into 2 pieces and then the “halved” bivalent and the two other bivalents detach from the bottom pole after the addition of NOC. Diagrams are included to clarify the laser cut and separation of “half” bivalents. Between **(a–e)** bivalents oscillate between the two poles, the position of one is indicated by the thin white arrow pointing to the kinetochore at the bottom pole. Two laser cuts were performed in quick succession; the first was not successful. The successful laser cut is shown in **(c)** a clear space is seen between the two pieces, indicated by the white bracket in **(d)** and **(e)**. The gap grows larger in **(e)**. The bottom “halved”-bivalent continued to oscillate, but stopped after several oscillations. The image went out of focus when NOC was added (in **g**). NOC caused the bivalents to stretch out, pause, detach from the bottom pole, then move quickly toward the top pole, as described by Fegaras and Forer, 2018a. The movement of kinetochores from the bottom pole is indicated by arrows pointing to kinetochores. The kinetochores (and attached bivalents or “halved” bivalents) stop moving once they near the top pole. The univalents remain behind in the bottom pole, as indicated by the white arrowhead in **(l)**. The cleavage furrow shifted toward the bottom pole. Time, as in all figures, is shown in mins:secs. **(B)** Graph of chromosome movement in the same cell, depicting the two kinetochores of an un-cut bivalent (red) and the two kinetochores of the two “half”-bivalents (blue). **(C)** Close-up of chromosome movement of both the cut and un-cut bivalents in the part of the graph indicated by the hashtag rectangle. Chromosome movement of un-cut bivalents follows the same pattern seen in NOC-only treated cells, which is bivalents stretch, pause, detach, fast speed, slow speed then stop. Bivalents that have been cut in two follow the same pattern, except there is no stretch phase because they are already positioned at their respective poles and there is no countervailing force toward the other pole from the previously-connected partner. The speeds of movement for the two moving kinetochores were determined from the slopes of the lines of best fit.

**FIGURE 4:**
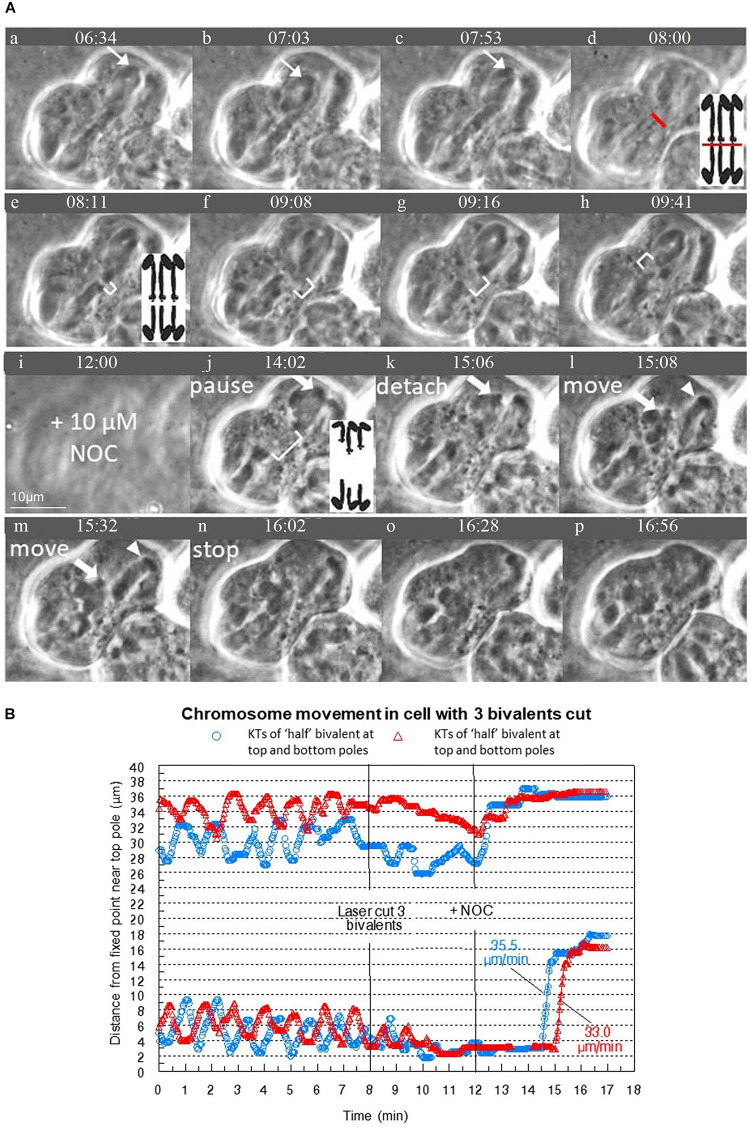
**(A,B)** Severed chromosomes are able to move after treatment with NOC when three bivalents are severed. **(A)** Image sequence of a cell where all 3 bivalents are severed as indicated by the red line. Diagrams are included to clarify the laser cut and separation of “halved” bivalents. In **(a–c)** the bivalents oscillate between either pole as shown by the thin arrows that point to a kinetochore. **(d)** is out of focus as the laser cut is performed along several places of focus. In **(e–h)** the cytosolic space is visible between the two pieces of the cut bivalents as shown by the white brackets. Throughout this time period, the bivalents oscillate irregularly, eventually stopping at their respective poles. The images of each of the frames is at a slightly different plane of focus to illustrate there is still a clear gap between all 3 of the severed bivalents, as also illustrated in the diagram. NOC was added at **(i)**. After the addition of NOC, the bivalents remain paused at both poles as seen in **(j)** (there is no “stretch” phase). They then detach, move quickly at first, then slowly toward the bottom pole, as shown by the thick white arrow, then stop near the bottom pole. The univalents do not move with the bivalents and remain at the top pole, as indicated by the arrowhead. The cleavage furrow moved toward the top pole. Time is shown in mins:secs. **(B)** Graph of the movement of four “halved” bivalents that were in the same plane of focus. The other two “halves” are in another plane of focus; their movements were not graphed but that the third bivalent was severed and that the “halves” moved to one pole were verified in the recorded image sequence in **(A)**. The speeds of movement for the severed kinetochores were determined from the lines of best fit.

We successfully cut bivalents in “half” in 30 cells. The resultant “halved” bivalents either moved to the poles and remained stationary there, or oscillated; the kinetochores of the oscillating halved bivalents moved toward and away from the pole they were associated with when they were part of a bivalent. The uncut bivalents continued their usual oscillation patterns after bivalents in the same cells were cut. The relative frequency of moving to the pole or continuing oscillating depended on the numbers of bivalents that were cut; the fewer bivalents that were cut per cell the more likely the “halved” bivalents would continue to oscillate (Table 1). In the cells in which more than one bivalent was cut, all “halved” bivalents acted the same: they either all oscillated or all moved directly to the pole. For all oscillating “half” bivalents, the initial oscillations were normal: they had the same velocities and periods as before being cut and the same as the continuing oscillations in not-cut bivalents. After varying numbers of normal oscillations, the oscillations first became irregular, with altered velocities and or periods, and then they ceased, as the “halved” bivalents became stationary at their poles. One such sequence is illustrated in Figure 3: as seen in the graph (Figure 3B, blue circles), the bottom “half” bivalent oscillated normally between 5 and 7 min, irregularly between 7–12 min, and then resided at the pole until NOC was added. The partner “half,” on the other hand (upper blue circles), oscillated normally from 5 to 14 min, then irregularly from 14 to over 15 min, and then was at the pole until NOC was added (at ∼17 min). It is typical that the partner “halves” do not necessarily follow the same pattern: sister kinetochores of the same bivalent (the two “halves”) generally stop oscillating at different times after the bivalent was cut. The one exception is one cell in which all 3 bivalents were cut: each of the “halves” oscillated irregularly for 3 cycles, and all stopped at their poles the same time (Figure 4).

**TABLE 1:** Numbers of cells with bivalent “halves” that continue to oscillate or become stationary at their respective poles.

Behavior of bivalent “halves”	Number of bivalents cut			Totals
	
	1 bivalent	2 bivalents	3 bivalents	
“halves” oscillate	12	4	3	19
“halves” stationary at the poles	3	2	6	11

**FIGURE 5:**
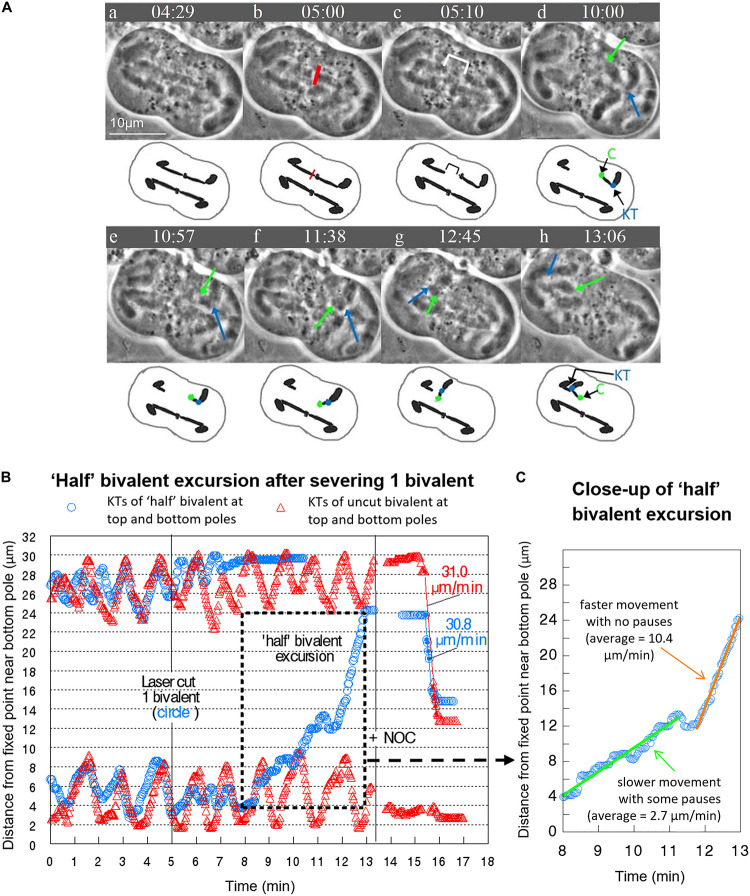
**(A–C)** Prior to addition of drug, severed “half” bivalents sometimes move to the opposite pole, similar to univalent excursions. **(A)** Video sequence of a cell with accompanying diagrams to clarify movement of the “half” bivalent. The red line indicates the laser irradiation of a single bivalent, with the cytosolic space between the two separated “halves” shown by the white bracket in **(c)**. In **(d)**, the bottom “half” bivalent is oriented with the chiasma (**C** – green dot and arrow) pointing toward the top pole, and the kinetochore (KT – blue dot and arrow) oriented toward the bottom pole. As the bottom “half” bivalent moves toward the top pole from e to g it rotates so that by **(h)** the kinetochore orients toward the top pole and the chiasma toward the bottom pole. In **(h)** the “half” bivalent reaches the top pole next to its partner “half” bivalent. For simplicity, we excluded from the diagram the univalent and the 1 bivalent that is not visible. Times are shown in mins:secs. **(B)** Movement graph of the excursion by a “half” bivalent in the same cell. The excursion begins at 8 min and reaches the opposite pole at 13 min. **(C)** Close up of the “half” bivalent excursion. There is a period of slower movement with some pauses (the line of best fit is green) followed by faster movement with no pauses (the line of best fit is orange). The average speeds of movement are taken from the lines of best fit.

Bivalent “halves” that first oscillated and then stopped moving remained at the poles, except in 3 cells in which only 1 bivalent was cut. In each of these cells the single “halves” first stopped at the poles and some time later moved across the equator to the opposite pole, which we refer to as “half” bivalent excursions. In all “half” bivalent excursions the kinetochores led the way. All three “half” bivalent excursions had two phases, initial slower movement and later faster movement. In the cell illustrated in Figure 5, for example, the “half” bivalent at the bottom pole finished oscillating (at 8 min on the movement graph, Figure 5B), was stationary at its pole for a short time, then began moving toward the opposite pole between 8 and 11 min, and then moved faster between 12 and 13 min (Figure 5C). For the 3 cells in which there were “half” bivalent excursions, the initial slower speeds averaged 3.3 μm/min. and the faster 7.6 μm/min.

#### Effects of Adding NOC to Cells With “Halved” Bivalents

In cells treated only with NOC (i.e., there was no laser cutting), all bivalents stretched, paused, detached from one pole, moved toward the opposite pole with a fast speed, slowed down as they reached the opposite pole, and stopped moving at the opposite pole [described in detail in Fegaras and Forer, 2018a]. In cells treated with NOC after bivalents were severed, at the same time that non-severed bivalents detached and move to the other pole the “halves” at the pole also detached and moved to the opposite poles, moving at the same time that the non-severed chromosomes moved, and at the same speeds as the non-severed chromosome kinetochores (see Figures 3B, 5B). The major difference in the behavior sequence after severing chromosomes is that the “halved” bivalents did not stretch out as the non-cut bivalents did, presumably because there was no force in the opposite direction otherwise supplied by the attached partner. As seen in Figure 3C, for example, the severed kinetochore (blue circle) is already paused at its respective pole when the not-severed kinetochore (red triangle) is in the stretch phase and both move to the opposite pole at the same time, and with the same speed. That the bivalent and “half”-bivalent movements are at the same speed suggests that the kinetochores move because of external forces, not those from tension in the stretched bivalent. We looked at these data in more detail to test how closely the speeds match.

#### Speed of Severed Bivalents Is the Same as That of Non-severed Bivalents

**FIGURE 6:**
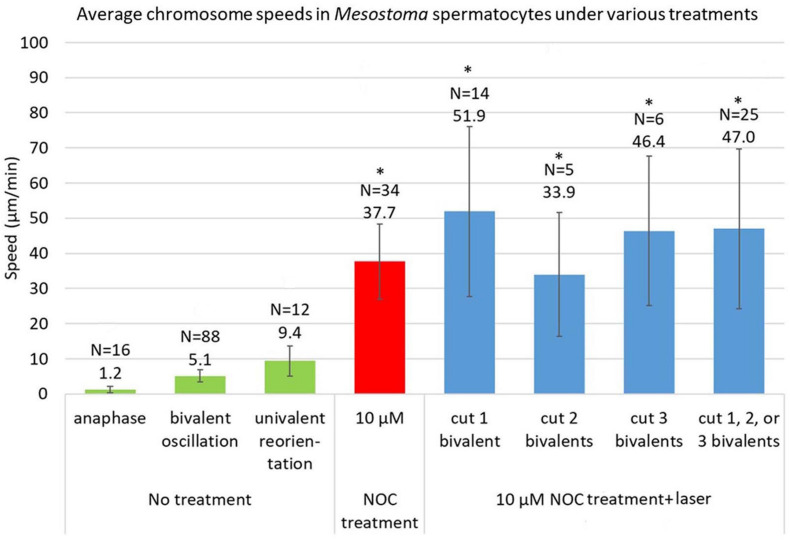
Average speeds of chromosomes in *Mesostoma* spermatocytes with no treatment, NOC-only treatment, and NOC + laser. The data for control cells (“no treatment”) is taken from Ferraro-Gideon et al., 2014. The speed of 10 μM NOC-only cells is an average of the fast speeds, and is updated from Fegaras and Forer, 2018a, to include data taken after that article was published. The data for NOC + laser cells include only cells in which bivalents or “halved” bivalents detached from 1 or 2 poles. The average speed of movement for NOC-treated cells *vs* NOC + laser treated cells are not significantly different. *represents values that are *not* significantly different, *p* > 0.05, as determined using Student’s *t*-test.

The total average speed of movement of “halved” bivalents in all cells with 1, 2, and 3 severed bivalents was not significantly different than the average speed of kinetochores in control cells (Figure 6). Furthermore, the speeds of the “halved” bivalents are very close to the speeds of the non-severed bivalents in the same cells (e.g., Figures 3B, 5B). Since “half” bivalents detached and moved to a pole at the same time and speed as non-severed bivalents in the same cell, and moved at the same speed as in control cells, the tension in the stretched chromosome does not contribute much, if anything, to the forces for movement. The forces for movement rather must arise from external non-microtubule forces. Other parameters of the movements of “halved” bivalents were not statistically different to those of non-cut bivalents, namely the time from NOC addition to detachment and the duration of the movements, regardless of whether 1, 2, or 3 bivalents were cut (Table 2 and Figure 6), so the absence of intact bivalents does not affect these parameters either.

**TABLE 2:** Timings and speeds of the chromosome responses for the different treatments.

Treatment *n* = number of cells	Time from drug addition to detachment (min:sec)	The total duration of kinetochore movement to the opposite pole, fast plus slow movement (min:sec)
10 μM NOC (*n* = 34)	02:06 ± 00:50 (01:07 03:44)	01:20 ± 00:51 (00:34–02:22)
Cut 1 bivalent + 10 μM NOC (*n* = 14)	02:04 ± 00:49 (01:01–04:20)	01:00 ± 00:35 (00:27–01:52)
Cut 2 bivalents + 10 μM NOC (*n* = 5)	01:27 ± 00:51 (00:29–02:29)	00:55 ± 00:19 (00:40–01:17)
Cut 3 bivalents + 10 μM NOC (*n* = 6)	01:44 ± 00:32 (00:59–02:22)	00:56 ± 00:25 (00:35–01:28)
TOTAL: Cut 1, 2, or 3 bivalent(s) + 10 μM NOC (*n* = 25)	01:52 ± 00:47 (00:29–04:20)	00:58 ± 00:29 (00:27–01:52)

**FIGURE 7:**
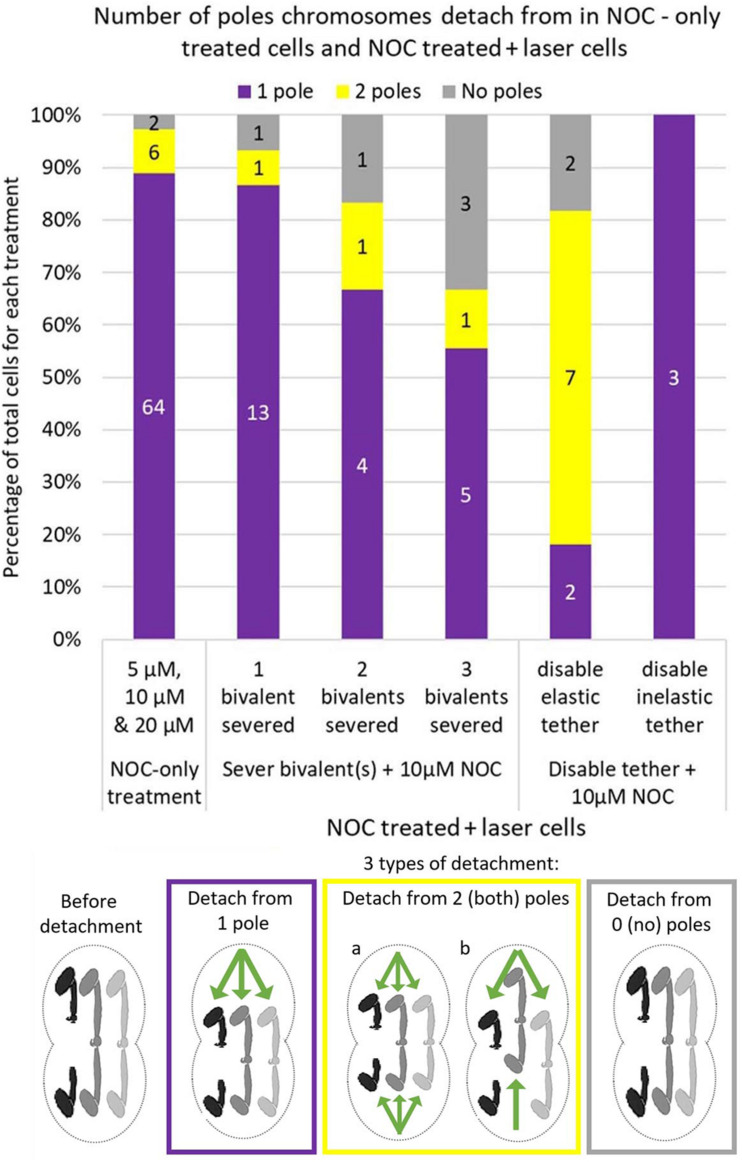
Cell numbers in which bivalents detach from 1, 2, or 0 poles in 5, 10, and 20 μM NOC-treated cells and 10 μM NOC-treated cells with 1 2 or 3 severed bivalent(s), or in 10 μM NOC-treated cells with disabled elastic or inelastic tethers. Data for NOC-only cells was updated from Fegaras and Forer, 2018a. Numbers in the middle of each bar indicate the actual number of cells in each condition, which is then converted to a percentage of total cells on the *y* axis. The diagram below the graph illustrates how the bivalents move when they detach from 1 pole, 2 (both) poles or 0 (no) poles. One bivalent (the black one) was cut in half for illustration purposes. Green arrows indicate the direction of movement of the kinetochores.

*Cell counts exclude 5 cells which had bivalent detachment from neither pole (see gray values in Figure 7 for number of cells with detachment from no poles). The values are ± standard deviations; the ranges are given in parentheses; the cell sample size is indicated by the *n* = value. None of the values in any given column are significantly different from others in the same column.*

We studied three additional issues, all dealing with coordination between chromosomes so that all detach at the same time, all detach from the same pole, and all move to the opposite pole.

#### After NOC Treatment, do “Half” Bivalents All Move to the Same Pole, as Bivalents do? the Likelihood of Detachment From Solely 1 Pole Decreases as the Number of Severed Chromosomes Increases

After addition of NOC to cells in which no bivalents were severed, the bivalents usually detached from one pole and the detached kinetochores moved to the other pole: they rarely detached from 2 or 0 poles (Figure 7). When bivalents detached from 0 poles, the bivalents stayed in the middle of the spindle, and no kinetochores moved toward either pole. When bivalents detached from 2 poles, either both kinetochores of each bivalent detached from both poles and all 3 bivalents were positioned in the middle of the cell, or bivalent kinetochores detached from 1 pole and moved to the other pole but different bivalents in the cell detached from different poles (see the diagram in Figure 7). After addition of NOC to cells in which bivalents were cut, the most common response was that “half” bivalents detached from one pole and moved to the other. As more bivalents were cut the likelihood “half” bivalents would detach from 1 pole decreased, and the likelihood they would detach from 2 or 0 poles increased (Figure 7). Thus, when one bivalent is severed, the “half” bivalents act as the bivalents do in control cells, but the coordination in detachments and in movements breaks down as more bivalents per cell are cut in half per cell. While this result may suggest that coordination in movements requires physical connection between “halved” bivalents, the physical connection that is required may be in the tethers that connect the free arms of the bivalent. Tethers physically connect the free chromosome arms and it may be that that physical connection is important for co-ordinating movements: some of the deleterious effects of severing bivalents on coordination between the chromosomes may be due to the laser inadvertently cutting tethers.

#### Tethers May Play a Role in Bivalent Detachment During NOC

**FIGURE 8:**
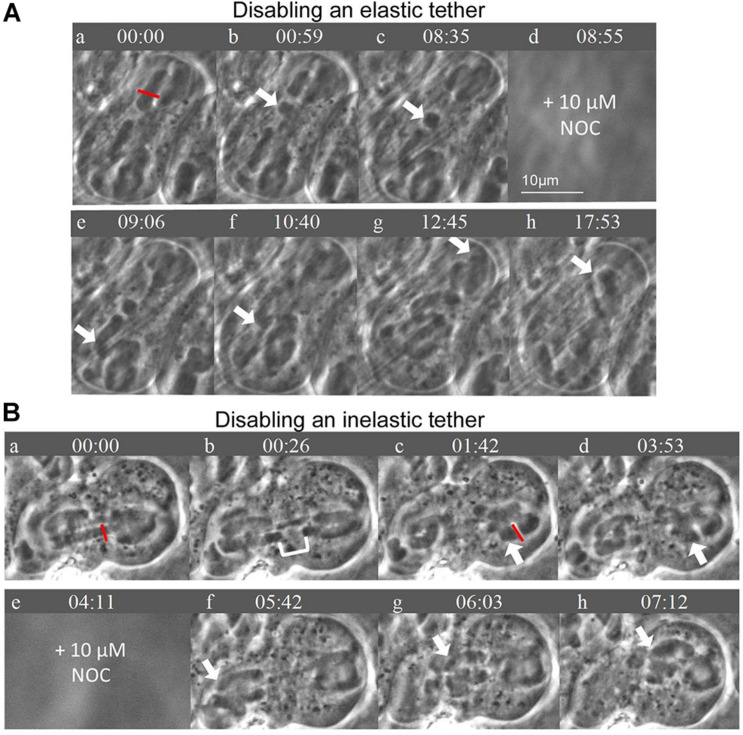
**(A,B)** Disabling elastic tethers connections affects bivalent detachment during the addition of NOC. Times are in min:sec. **(A)** After disabling an elastic tether, chromosome movement is altered in that after subsequent treatment with NOC, all bivalents detach from both poles. An arm fragment was severed using the laser as indicated by the red line. The arm fragment moves backwards across the cell equator toward the opposite bivalent arm, as indicated by the white arrows. That the arm fragment moves indicates that the tether is elastic; that the arm fragment is disconnected from the arm means the tether has been disabled. After the addition of NOC, all bivalents detach from the bottom pole (**e** and **f**) as indicated by the white arrows; subsequently all detach from the top pole (**g** and **h**). **(B)** After disabling an inelastic tether, bivalent detachment is not altered in the presence of NOC. First one bivalent is cut in half using the laser, as indicated by the red line; the cytosolic space between the separating “half” bivalents is shown by the white bracket in **(b)**. Then on the same bivalent, one of the arms in the right half-spindle is cut, indicated by the red line **(c)**. As shown by the white arrow in **(c)** to **(d)** the arm fragment does NOT move backwards across the cell equator, indicating the tether was either inelastic, or was not present, or was accidentally severed when the bivalent was severed. After the addition of NOC, chromosomes move with the expected pattern of movement: all bivalents detach from the left pole and move toward the right pole, as indicated by the white arrows in **(f–h)**. There are no chromosomes left behind at the left pole in frame **(h)**.

Elastic tethers connect the telomeres of all separating anaphase chromosomes in a variety of animal cells: the arm fragment cut from one of the arms moves across the equator to the telomere of the partner chromosome (LaFountain et al., 2002; Forer et al., 2017). As summarized in the Introduction, “tethers” connect separating anaphase chromosomes in a broad range of cells, including *Mesostoma* spermatocytes, and would seem to be present universally in animal cells (Forer et al., 2017). Anaphase “tethers” are elastic in early anaphase but become inelastic as they elongate. Tethers also extend between the telomeres of the free arms of *Mesostoma* spermatocyte bivalents during prometaphase: after severing a portion of the free arm, the arm fragment moves to the telomere of the other free arm (Forer et al., 2017). But not all the time, only in an estimated 50% of all cases (Fegaras-Arch, unpublished). Since tethers produce tension between the arms, we thought that tension from tethers might be involved in determining the detachment of the kinetochores, e.g., in co-ordinating kinetochores so that only one kinetochore detaches, and co-ordinating which pole it detaches from. To test the role of tethers we disabled them directly by cutting a section from the tip of a free arm [Figure 2B, Laser experiment]; this disconnects the mechanical connection between the arms and hence to the kinetochore to which it originally was attached. Since tethers cannot be visualized, one needs to cut arms to test if an elastic tether is present (Paliulis and Forer, 2018). If elastic tethers are present, the arm fragment moves to the partner telomere (LaFountain et al., 2002), as illustrated in the cartoon in Figure 2B. In the cell shown in Figure 8A, one of the free bivalent arms at the top pole was severed and the arm fragment moved backwards across the equator toward the telomere of the opposite arm, nearer the bottom pole. In the cell shown in Figure 8B, on the other hand, the arm was severed but the arm fragment did not move backwards, indicating that either a tether was not elastic or it was not present. In our experiments, we severed tethers of one bivalent, added NOC, and asked if bivalent behavior was altered. When we disabled *elastic* tethers and treated those cells with NOC, bivalent detachment was altered: there was increased frequency of cells in which bivalents either detached from both poles or did not detach from either pole (Figure 7). In comparison, when we severed *inelastic* tethers, bivalent behavior was normal: in 3/3 cells all bivalents detached from 1 pole and the detached kinetochores moved to the other (Figure 7). This experiment points to elastic tethers having an important role in co-ordinating the movements of bivalent kinetochores so that they all move to the same pole (after treatment with NOC), that tethers must physically connect and put tension on free arms in order for the coordinated after-NOC movements to occur.

#### “Half” Bivalent Oscillations Predict Which Pole Kinetochores Will Detach From

It is a puzzle that after treatment with NOC all bivalents detach from one pole at the same time, and all kinetochores move to the opposite pole. None of the features of cell division in *Mesostoma* spermatocytes that we have looked at have allowed us to predict which pole the kinetochores will detach from (Fegaras and Forer, 2018a), but detailed analysis of the “half” bivalent movements seem to indicate that there might be differences between the two spindle poles. We compared the behavior of “half” bivalents at the two poles, the pole from which all kinetochores detached after NOC was added vs that at the pole where kinetochores did not detach. The parameters we looked at are listed in Table 3. Two of the parameters indicated which pole that kinetochores would detach from, the “half” bivalent that stopped oscillating first, and the length of time the “half” bivalents oscillated (normal plus irregular oscillations). At the pole from which kinetochores subsequently detached, the “half” bivalent stopped oscillating before the partner “half” bivalent did in 12/15 cells (illustrated graphically in Figure 3B), and the duration of oscillations was shorter than that of the partner “half” bivalent (averaging 3 min:35 s vs 4 min:57 s).

**TABLE 3:** Comparison of “half” bivalents oscillations at the pole that DID detach, vs. DID NOT detach.

	“Half” bivalents at pole from which			“Half” bivalents at pole from which		
	kinetochores subsequently DID detach			kinetochores subsequently DID NOT detach		
		
Laser cut(s)	Average # of normal oscillations	Average # of irregular oscillations	Average duration of normal plus irregular oscillations (min:sec)	Average # of normal oscillations	Average # of irregular oscillations	Average duration of normal plus irregular oscillations (min:sec)
Cut 1 bivalent (*n* = 12)	2.3 ± 1.9	2.2 ± 1.2	03:35 ± 02:01*	3.0 ± 1.7	2.4 ± 1.0	04:57 ± 03:01*
Cut 2 bivalents (*n* = 4)	0	2.5	–	2.5	1	–
Cut 3 bivalents (*n* = 3)	1	3	–	1	2	–

Data in this table is only of the cells tabulated in the “‘halves’ oscillate” row in Table 1, excluding cells with “bivalent ‘halves’ stationary at the poles”. The ± standard deviations are included where applicable. Values with *are significantly different at *p* < 0.05 using Students *t*-test. The average duration of normal plus irregular oscillations is statistically greater for “half” bivalents at the pole from which there was no detachment, but there appears to be no significant difference between the numbers of normal oscillations, or of the numbers of irregular oscillations.

## Discussion

### Force to Move Chromosomes

A main conclusion from our experiments is that after removal of spindle microtubules with NOC (Fegaras and Forer, 2018a) “halved” bivalents move with the same rapid speeds as normal bivalents in the same cell, Therefore, rapid kinetochore movement in the absence of spindle microtubules is not due to tension in the stretched bivalents and must be due to some spindle component(s) other than microtubules. What could the force producers be?

In the presence of 10 μM NOC, chromosomes in *Mesostoma* spermatocytes with no severed bivalents stretch and then selectively detach from one pole and move toward the opposite pole at speeds that average 37.7 μm/min. (Figure 6 and Fegaras and Forer, 2018a). Experiments presented here show that this movement is not due to recoil of stretched chromosomes because when bivalents are cut, the “halved” bivalents still detach from one pole and move toward the opposite pole, and they do so at an average speed of 47.0 μm/min. (Figure 6). There does not appear to be a statistically significant difference in the average speeds for cells treated with 10 μM NOC without cutting bivalents vs after cutting 1, 2, or 3 bivalents (*p* > 0.05). Since the “half” bivalents and the non-cut bivalents move at the same (or very similar) speeds, this indicates that similar forces act on the kinetochores of the moving “halved” bivalents and of the full bivalents, and that the forces do not derive from tension in the stretched bivalents. Nor is the force for these kinetochore movements from microtubules, because immunofluorescence staining shows that NOC treatment removes microtubules from the spindle: most spindle microtubules are depolymerized by the NOC. Any that remain are highly fragmented and not attached to the pole, and none are in contact with the moving chromosomes (Fegaras and Forer, 2018a). Since no microtubules are present, the movement is due to some force different from microtubules.

We suggest that what may be moving the chromosomes is actin and myosin, perhaps as components of the spindle matrix (Johansen et al., 2011). In the spindle matrix model, microtubules play a passive role in chromosome movement, acting as governors rather than force producers (Spurck et al., 1997; Johansen and Johansen, 2007; Pickett-Heaps and Forer, 2009; Johansen et al., 2011). What may instead be providing the force for chromosome movement is actin, myosin, or some other spindle matrix proteins (Fabian and Forer, 2007; Fabian et al., 2007; Forer et al., 2015). We can test this hypothesis by using various enhancers and inhibitors that target actin and myosin in NOC-treated cells. If perturbing these proteins alters chromosome movement, that would support our suggestion that actin and myosin play a role in the chromosome movements that occur after NOC treatment.

### Coordination Between Chromosomes: Detachment From One Pole

In NOC treated cells all 3 bivalents detach from one pole, almost always the same pole, and all three kinetochores move rapidly to the other pole (Figure 7). Halved bivalents act the same way: most detach from one pole (Figure 7), always the same pole that the un-cut bivalents detached from (when there were fewer than 3 halved bivalents in the cell). Our data suggest that elastic tethers are important for these coordinations. After severing arms in *Mesostoma* spermatocytes, the arm fragments sometimes move to their partners, and sometimes they do not (also noted by Ferraro-Gideon, 2013), indicating that sometimes there are elastic tethers between the arms, and sometimes tethers either are not present or are inelastic. In our experiments, in cells in which we disabled an elastic tether, bivalents had an increased frequency of detachment from 2 poles after treatment with NOC (Figures 7, 8A). Severing an arm that did not have an elastic tether, on the other hand, did not alter chromosome detachment from 1 pole after addition of NOC (Figures 6, 8B). We think that tethers produce tension between the two free arms of each bivalent, and that this tension may be involved in co-ordinating chromosome movement to one pole after treatment with NOC. If tethers are involved in this way, this is consistent with the role of tethers found in crane-fly spermatocytes, in which tethers appear to be involved in co-ordinating movements of separating anaphase chromosomes (Sheykhani et al., 2017; Paliulis and Forer, 2018; Forer and Berns, 2020).

Damage to tethers may be the reason that cutting more bivalents per cell results in reduced coordination: as more bivalents are cut the more likely it is that coordination is altered and that halved bivalents do not all go to the same pole (Figure 7). We suggest that this is because the likelihood of accidentally cutting a tether increases as one severs more bivalents because that requires several larger cuts in various focal planes. It is tricky to avoid cutting tethers even when cutting 1 bivalent in “half,” and it is very difficult to avoid cutting tethers when cutting 2 or 3 bivalents, all in several focal planes. Therefore, the decrease in detachment from 1 pole may be due to the fact we accidentally cut elastic tethers while cutting 2 or 3 bivalents in the same cell.

*Mesostoma* spermatocytes so far are unique in having tethers between chromosome arms prior to anaphase, having been found between the ends of the free arms in prometaphase. In other cells, in early anaphase tethers are elastic; in later anaphase the longer tethers are inelastic (Forer et al., 2017), and in crane-fly spermatocytes, at least, the difference seems to be coordinated with phosphorylation and dephosphorylation events (Kite and Forer, 2020). Tethers in *Mesostoma* may be similar: it is possible that *Mesostoma* tethers alternate between phosphorylation states, or perhaps not all tethers are phosphorylated in any one given cell, and that is why sometimes severed arm tips might not move.

### Coordination Between Chromosomes: Oscillation of “Halved” Bivalents as Markers for Which Pole the Kinetochores Will Move to After NOC Treatment

“Halved” bivalents usually continued to oscillate for some period of time after the bivalents were cut in two by the laser. In general, partner “halved” bivalents start by oscillating regularly, then they oscillate irregularly, and then they stop at their respective poles (Figures 3B, 4B, 5B), usually at different times: one “halved” bivalent generally stops several oscillations before its partner. When kinetochores subsequently detach from one pole and move to the other, they usually (12/15 cells) move from the pole at which “half” bivalent oscillations stopped first. The detailed timings shown in Table 3 confirm that the duration of both normal and irregular oscillations was significantly shorter at the pole from which kinetochores DID detach. This suggests that some difference in the spindle forces at that pole plays a role in chromosome detachment from that pole. We don’t know, however, whether the difference arises from something inherent in that particular one of the 2 poles, or rather whether there are inherent differences in the 2 “half” bivalents (e.g., male-derived vs female-derived).

### Other Issues

That the “half” bivalents continue to oscillate counters the argument by Fuge about the mechanisms of the regular oscillations of bivalents in prometaphase. Fuge (1987) argued that the normally-occurring bivalent oscillations arise from forces from opposite poles transmitted by tension in the chromosomes: the pulling forces from the spindle fibers were countered by tension along the chromosome and that this interplay caused the normal oscillations. Our experiments counter this argument since “halved” bivalents still oscillate, at least for a while. Thus, the forces for the continuing oscillations are not from opposite kinetochores but must arise from something inherent in the forces produced by the spindle.

Why do regular oscillations stop after several cycles of normal and then irregular oscillations begin (Table 3)? While the lack of tension along the length of the “halved” bivalents may eventually stop the oscillations, there may be another contributing factor. Prometaphase bivalents often go through periods where their oscillations shift in phase; graphically these look similar to the irregular oscillations of a severed “half” bivalent (e.g., Figures 6, 7 in Ferraro-Gideon et al., 2014; Figure 7B in Fegaras and Forer, 2018a). Phase shifts may or may not occur in control cells in any given sequence we film, and they can occur multiple times to any one bivalent at anytime throughout prometaphase. It is possible that whatever mechanisms are involved in causing phase shifts, and that take place during a phase shift, are also at work during the period of dampened oscillations in “halved” bivalents.

“Half” bivalents eventually pause at the poles after oscillating. Some of them then move across the spindle to the opposite pole (Figures 5A–C). During “half” bivalent excursions the kinetochore of one “half” bivalent detaches from a pole, swings around toward the opposite pole, and moves toward the opposite pole, in a similar manner to univalent excursions in *Mesostoma* (Ferraro-Gideon et al., 2013, 2014), and to sex chromosomes in other cell types such as grasshopper spermatocytes (Nicklas, 1961; Ault, 1984, 1986) and crane-fly spermatocytes (Bauer et al., 1961). We do not know what controls these movements. Excursions of univalent chromosomes from pole to pole seem to be needed to obtain proper distributions of the two pairs of univalent chromosomes (Oakley, 1985), so *Mesostoma* spermatocytes indeed have mechanisms to aid pole-to-pole movements. Though we have no idea what they are, it is not surprising that they act on “halved” bivalents that are at the poles together with the univalent chromosomes. Since anaphase seems to be inhibited until proper distribution of univalents is obtained at the poles, it may be that “halved” bivalents inhabiting a pole may trigger the movement to the opposite pole through whatever mechanism triggers the univalent chromosomes to move. Since improper distribution of univalents seems to inhibit anaphase onset (Oakley, 1985) it may be that the presence of “halved” bivalents at the poles inhibits anaphase. This is speculation on our part, and further testing is needed.

### In Summary

One main conclusion from our experiments is that non-microtubule spindle components can move chromosomes in *Mesostoma* spermatocytes. After microtubules are removed with NOC, all chromosome kinetochores move at rapid speeds (up to 100 μm/min) to the opposite poles. Our experiments have eliminated tension in elongated chromosomes as producing these forces since the same movements occur in “halved” bivalents which do not have bipolar connections. Chromosome movements still occur when 1, 2, or 3 bivalent(s) are severed into “halved” bivalents, and the movements are at very close to the same rapid speeds as in not-cut bivalents. A second conclusion is that elastic tethers may be required to coordinate kinetochore movements. Cutting more than one bivalent per cell alters the coordination by which all kinetochores release from one pole and move to the opposite pole. Because directly disabling one elastic tether in a cell alters the coordinated movements in that cell, we suggest that the altered coordination in moving to one pole when all bivalents are severed is because of collateral damage to tethers when cutting multiple bivalents.

## Data Availability Statement

The raw data supporting the conclusions of this article will be made available by the authors, without undue reservation.

## Author Contributions

EF-A conducted all original experiments, analyzed the data, created the figures and tables, and wrote the manuscript. EF-A, AF, and MB edited manuscript drafts and AF contributed to planning the experiments. The experiments were done in the lab of MB. All authors contributed to the article and approved the submitted version.

## Conflict of Interest

The authors declare that the research was conducted in the absence of any commercial or financial relationships that could be construed as a potential conflict of interest.
